# Respiratory illness virus infections with special emphasis on COVID-19

**DOI:** 10.1186/s40001-022-00874-x

**Published:** 2022-11-08

**Authors:** Lekha Gandhi, Deepti Maisnam, Deepika Rathore, Preeti Chauhan, Anvesh Bonagiri, Musturi Venkataramana

**Affiliations:** Department of Biotechnology and Bioinformatics, School of Life Sciences, University of Hyderabad, Hyderabad, 500046 Telangana India

**Keywords:** COVID-19, Respiratory virus infections, Influenza viruses, SARS, MERS

## Abstract

Viruses that emerge pose challenges for treatment options as their uniqueness would not know completely. Hence, many viruses are causing high morbidity and mortality for a long time. Despite large diversity, viruses share common characteristics for infection. At least 12 different respiratory-borne viruses are reported belonging to various virus taxonomic families. Many of these viruses multiply and cause damage to the upper and lower respiratory tracts. The description of these viruses in comparison with each other concerning their epidemiology, molecular characteristics, disease manifestations, diagnosis and treatment is lacking. Such information helps diagnose, differentiate, and formulate the control measures faster. The leading cause of acute illness worldwide is acute respiratory infections (ARIs) and are responsible for nearly 4 million deaths every year, mostly in young children and infants. Lower respiratory tract infections are the fourth most common cause of death globally, after non-infectious chronic conditions. This review aims to present the characteristics of different viruses causing respiratory infections, highlighting the uniqueness of SARS-CoV-2. We expect this review to help understand the similarities and differences among the closely related viruses causing respiratory infections and formulate specific preventive or control measures.

**Supplementary Information:**

The online version contains supplementary material available at 10.1186/s40001-022-00874-x.

## Introduction

**Fig. 1:**
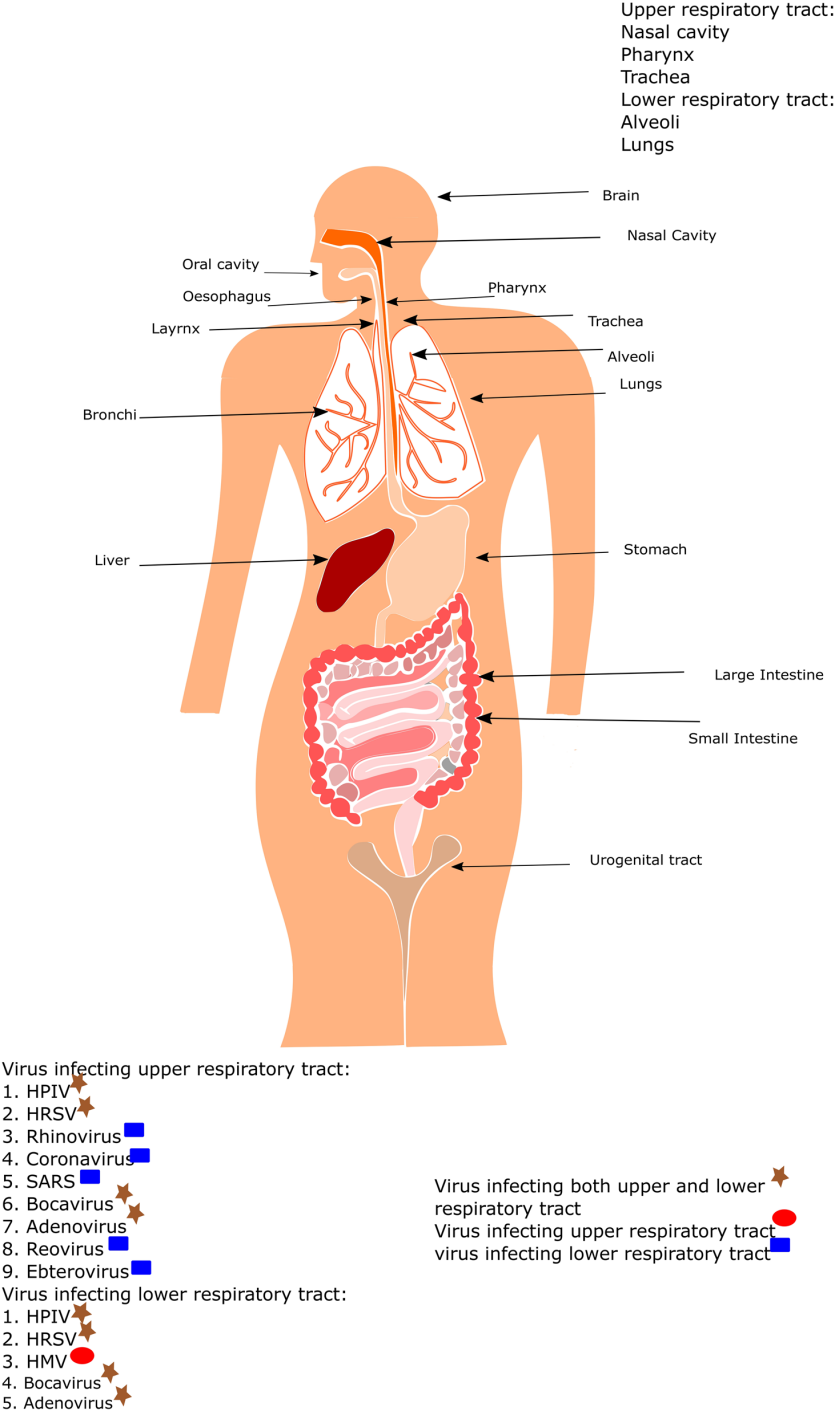
Diagrammatic representation of the human body showing the entry ports for different respiratory disease-causing viruses. The preferential binding site for all the viruses is trachial ciliated epithelial cells. HPIV (Human Parainfluenza virus), HRSV(Human respiratory syncytial virus), SARS (Severe Acute Respiratory syndrome), HMV (Human Metapneumovirus)

**Fig. 2:**
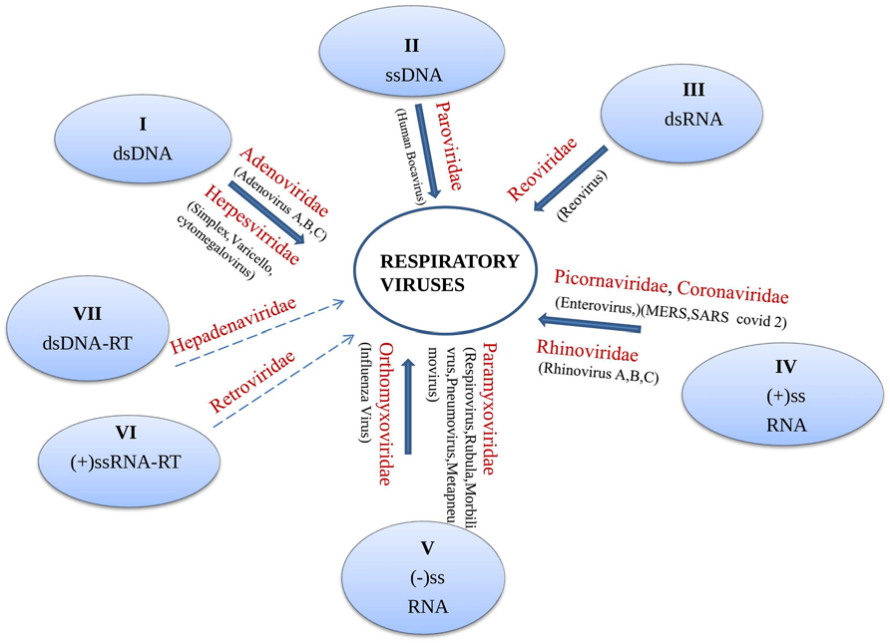
Taxonomic status of the respiratory viruses in Baltimore system of classification (continuous arrow indicates the virus family that contains the respiratory virus(s), whereas discontinuous arrow indicates that the family does not contain any respiratory virus). dsDNA (double stranded DNA), ssDNA (single stranded DNA), dsRNA (double stranded RNA), (+) ssRNA (positive sense single stranded RNA), (−) ssRNA (negative sense single stranded RNA), ssRNA-RT (single stranded RNA reverse transcribing), dsDNA-RT (double stranded DNA reverse transcribing)

Studies on viruses and viral diseases have been carried out in science, medicine, and agriculture for hundreds of years, leading to the emergence of virology as a separate branch of microbiology. Viral epidemics and pandemics occur periodically throughout the world. Virologists have contributed well to virus research to understand the structural organization, cellular mechanisms, vaccination, and antiviral molecules against many viruses. In the 1900s, WHO eradication efforts of some viruses such as smallpox, cowpox, measles, rabies and poliomyelitis brought significant advancement in controlling and preventing the diseases and understanding the viral replication, transmission, pathogenesis, cellular functions and disease manifestation. Despite large diversity, they share common principles to enable the infection. Another conserved feature between viruses is activating cell signalling processes when interacting with cells [1]. In this direction, studies are required to decipher the characteristics of many viruses causing diseases [2]. Viruses infect almost all living organisms, including bacteria, fungi, fish, birds, plants, animals, and humans. There are different ways of virus transmission, such as vector borne, blood borne, aerosol borne, etc. [3]. The compatible receptors mostly decide this host range on the tissues or the cells [4]. Similarly, within the host organ/tissue, tropism is also directed by the presence of specific receptors for different viruses. As another aspect of viruses, the Baltimore system of classification of viruses divides the viruses into seven different classes based on the messenger RNA (mRNA) formation from the parental genome [5]. They are 1. Double stranded DNA (dsDNA), 2. Single stranded DNA (ssDNA), 3. Double stranded RNA (dsRNA), 4. Positive sense single stranded RNA (ssRNA + ve), 5. Negative sense single stranded RNA ( ssRNA −ve), 6. dsDNA with reverse transcriptase (RT) and 7. Single stranded RNA (ssRNA) with Reverse Transcriptase (RT). There are 12 different viruses belonging to different virus families that cause infections in the respiratory system (Fig. 1; Additional file 1: Table S1) [6–10]. At least one virus from each of the virus family of the Baltimore virus classification represents respiratory infections (Fig. 2) [5]. These viruses are widely diversified in their genetic makeup and exist in multiple strains. They infect human, animals and birds and cause the similar symptoms, such as fever, cough, sneezing, diarrhea etc. The viruses with RNA as their genome undergo frequent mutations due to the less proofreading activity of their replicase enzyme, i.e., RNA-dependent RNA polymerase (RdRp). The majority of the viruses causing respiratory infections are with the RNA as their genome and tend to attain the mutations in the genome of the progeny virus. In the case of respiratory infections, nearly all the viruses transmit by droplets, person-person contact or by fomites.

History indicates the occurrence of pathogenic pandemics or epidemics from time to time. Plague, Spanish flu, Smallpox, SARS-CoV, MERS-CoV, Ebola and presently SARS-CoV2 causing COVID-19 represent the best examples [11]. Millions of people were affected and lost their lives during all the above occasions. Influenza, respiratory syncytial, parainfluenza and metapneumo viruses are the four major viral pathogens associated with acute lower respiratory infections. These represent a substantial disease burden, particularly in young children (˂5 years) and older people (˃65 years). Globally, the influenza virus is estimated to cause 39.1 million acute lower respiratory infections and 58,200 deaths annually, and the respiratory syncytial virus is estimated to cause 24.8 million infections and 76,600 deaths annually [12]. In addition, members of the *herpesviridae* family that include herpes simplex, cytomegalovirus, varicella-zoster virus, Epstein–Barr virus and human herpesvirus type 6 (HHV6), have been associated with respiratory diseases [13]. Mean R_0_ of influenza is 1.68, RSV 3.47, rhinovirus 1.88 coronavirus 4.18 and adenovirus 2.34. This indicates the need for serious attempts for their control [14]. Furthermore, these viruses are responsible for greater annual morbidity and mortality than influenza viruses across all age groups [15]**.** In light of the above information, a comprehensive analysis of respiratory virus infections with particular emphasis on COVID-19 is given below, which might help better understand and formulate the precise control measures at appropriate stages of outbreaks.

## Adenoviridae

### Human adenovirus

A transmissible agent was first isolated in 1953 from adenoid tissue showing cytopathic effect. In 1956, the name ‘adenovirus’ was proposed for this new respiratory tract virus [16]. This human-adenovirus (HAdV) belongs to the family *Adenoviridae* and genus *Mastadenovirus*, which is further classified into seven types of species HAdVs (A–G). Among them, the species that mainly cause respiratory tract infections are B, C and E [17]. Currently, the distribution of adenovirus is worldwide but was primarily attributed in Europe, North America, Taiwan, China, Japan, Singapore and Russia during the late 1950s and early 1960. The serotype of species C was more predominant during this period, followed by the prevalence of B and E. More number of affected people showed respiratory infection symptoms, followed by a few with conjunctivitis. Brandt and colleagues did the first long-term study on a huge random population in 1969 by pooling 18,000 children for 10 years and studying the symptoms of adenovirus [16]. Later on, in the following years, many reports from individual countries were reported for the seroprevalence of adenovirus in children as well as in immunocompromised adults. Immunosuppressed individuals are found to be at risk of death from an adenovirus (AdV) infection. More than 80 different AdV types (within the A to G species) infect humans through the respiratory, ocular, or gastrointestinal tracts. Approximately 70 serotypes were classified by hemagglutination, serum neutralization reactions, genomic sequencing and bioinformatic analysis. A study conducted by the National Adenovirus Type Reporting System (NATRS), a USA military camp from 2003 to 2016, indicated that serotypes 1, 2, 3, 4, 7 and 14 were the predominant cause of infection [18]. In its latest global survey, WHO has released that 20% of adenoviral infections are caused by serotypes 7 and 14 [19]. The predominant HAdV types can change over time within a region and transmission of new strains across continents appears frequent.

**Fig. 3:**
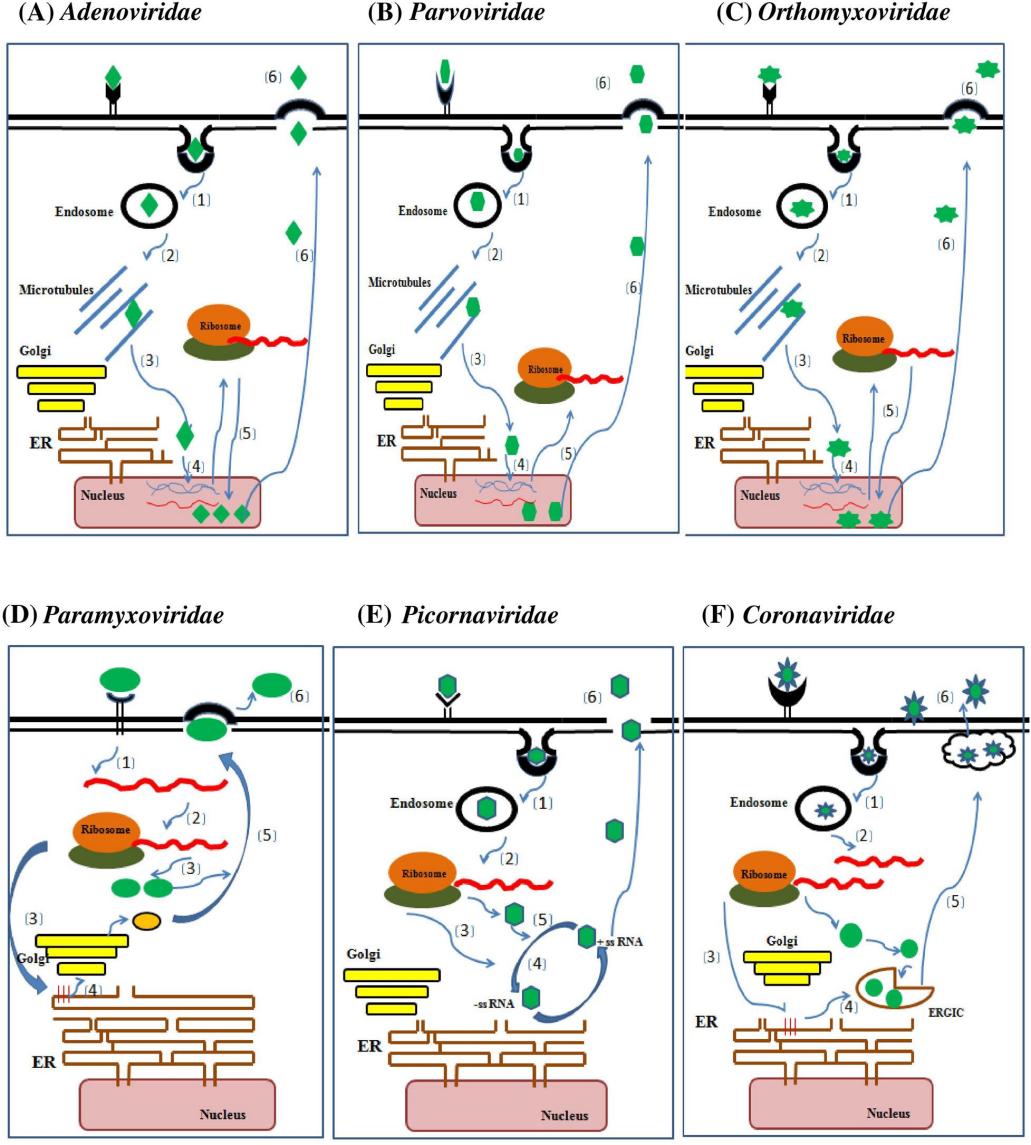
Diagrammatic representation of the life cycles of the viruses causing respiratory infections. The life cycle includes 1. Endocytic uptake of virus inside the cell, 2. Release of virus from Endosome in cytosol, 3. Microtuble mediated Trafficking of viral particle 4. Uncoating and Release of viral genome into the nucleus for further replication, 5. Viral particles assembled in the nucleus and 6. Cell lysis and release of new virions outside cell. [(Viruses of the families **A**, **B** and **C** enters the cell by binding through cell receptors (CAR for *Adenoviridae*, VP1u-interacting protein for *Parvoviridae*, S for *Orthomyxoviridae).* Internalization takes place by receptor mediated endocytosis inside the cells encapsulated by endosome. The internalised virus is further released from endosome and partially uncoated virus will be directed to nucleus through microtubule network. The virus disassembly occurs in nucleus. The viral genome will be released and replicates inside the nucleus. The transcribed mRNA will be translated in cytosol and expressed proteins will be imported to nucleus, where new virus assembly takes place. Newly assembled virus particles will be released outside the cell by cell lysis]. Whereas the viruses of the families **D**, **E** and **F** binds to the cells through cell surface receptors but *Paramyxoviridae* will be released directly into cytoplasm, whereas *Coronaviridae* and *Picornaviridae* will be internalised inside endosome and released in cytoplasm by endosome lysis. In *Paramyxoviridae*, following release of negative sense RNA genome, positive sense RNA will be produced for protein synthesis. Whereas in *Picornaviridae* and *Coronaviridae,* the positive sense RNA genome is replicated into intermediate –ve sense RNA which then serves as template to form positive sense RNA genome of the progeny virus. The assembly of viral particles takes place at ER-Golgi intermediate compartment but in *Paramyxoviridae* and *Picornaviridae* virus assembly takes place at cytosolic double membrane compartment and plasma membrane, respectively. Nascent virion budding takes place through plasma membrane in *Paramyxoviridae* but in *Coronaviridae* the virus is transported in outer space through exocytic vesicles. In *Picornaviridae* the newly synthesized virus is directly released through cell lysis. (ER: endoplasmic reticulum)

The adenovirus genome is a double-stranded ~ 24–46 kb size DNA, enclosed by an outer capsid and an inner nucleoprotein core complex. The capsid has icosahedral symmetry and the virion is non-enveloped, measuring up to 90 nm in diameter [20]. This dsDNA codes for various early (E) and late (L) proteins, which express before and after viral replication (Fig. 3A). HAdVs cause infections in the respiratory, digestive and ocular tracts. Transmission takes place by the route of aerosolized droplets by airborne, fecal contact with the infected or contaminated surfaces. The virion infects the host cell by interacting with the receptors coxsackie adenovirus receptors (CAR), membrane cofactor protein (MCP) and desmoglein-2 present on the host cell, thereby initiating the respiratory infection [21, 22]. Once the replication process starts, the virus infects the macrophages, which trigger the inflammasome, causing inflammation of the lungs leading to pneumonia. The fever is caused by the innate immune response [23]. The adenovirus infection ranges from mild to severe conditions for which the incubation period is generally less than 10 days. All age groups are susceptible to this virus infection. The symptoms seen in adenoviral diseases, caused by species B, C and E, include respiratory tract infections, such as pneumonia, fever, worsening cough, diarrhea, vomiting, and lethargy. This virus is an important cause of infections in immunocompetent and in immunocompromised hosts causing mild upper respiratory infections (pharyngitis, coryza, conjunctivitis) and complicated infections (pneumonia, hepatitis, hemorrhagic cystitis or disseminated diseases), respectively. Adenoviral pneumonia is another clinically important entity, and serotypes 3, 7, 14, 21 and 55 have been associated with severe and complicated presentations. In immunocompromised adults and infants, pneumonia may worsen and the patient might require mechanical ventilation, and death may occur in terminal cases [19].

Diagnosis of the HAdV is mainly done by Computed Tomography (CT) scan in severe cases. In mild cases, the diagnosis is by direct fluorescent assay, virus culturing and Polymerase Chain Reaction (PCR)-based techniques. The study of adenovirus is vital to find a cure for it and further to understand the molecular mechanisms underlying the infection cycle of the virus as adenovirus has been used as a vector since the late 1980s. As of now, there are no available vaccines for HAdV to the general public, but the US Food and Drug Administration (FDA) in March 2011, approved the use of a vaccine only for military personnel against serotypes 4 and 7 [19]. Research on antiviral drugs such as histone–deacetylase–inhibitor–SAHA, ganciclovir and cidofovir is ongoing [24, 25]. Along with the antiviral drugs, many biological compounds are being synthesized as biological antivirals, including RNAi, T-cell therapy, etc. [26].

## Parvoviridae

### Human bocavirus

Tobias Allegender and co-workers reported the human bocavirus (HBoV) for the first time in 2005 at Karolinska University Hospital, Stockholm, Sweden. The virus was isolated from the nasopharyngeal secretion of a pediatric patient infected with a lower respiratory tract infection [27]. HBoV is increasingly being distributed worldwide and infects throughout the year but primarily in the months of winter and spring. Human bocavirus is a causative agent of respiratory infection; a non-enveloped, single-stranded 5.3 kb sized DNA virus belonging to the genus *Bocaparvovirus*, sub-family *Parvovirinae* and family *Parvoviridae*. The size of the virions is approximately 18–26 nm and exhibits icosahedral symmetry [28]. Bocavirus comprises the bovine parvovirus 1 (BPV1), minute virus of canines (MVC) and a human bocavirus (HBoV). HBoV has four species (HBoV 1–4) based on the divergence of the VP1 gene nucleotide region [29–31]. HBoV is known for upper and lower respiratory tract infections, sometimes as a single agent or co-pathogen with other viruses.

The viral genome contains three open reading frames (ORFs) in which ORF1 and ORF2 encode two non-structural proteins, i.e., Non Structural Protein 1 (NS1) and Nuclear Phospho Protein (NP1) and ORF3 encode structural capsid proteins of viral particle viz ( VP1, VP2 and VP3) [32]. Alternatively, spliced mRNA transcripts process the non-structural proteins NS1, NS2, NS3, NS4 and the above mentioned non-structural proteins are not essential for the expression of capsid proteins. NS1 and NS1–70 proteins block the activation of Nuclear factor–Kappa B (NF-KB) in the presence of Tumor Necrosis Factor alpha (TNF-α) response [33]. Nuclear Phospho protein is a highly phosphorylated non-structural protein and NP1 plays a crucial role in the expression of capsid protein and helps in splicing of mRNA. In the absence of NP1, the expression level of HBoV1 capsid proteins is effectively very less. This viral NP1 protein of HBoV1 and minute virus of canine are similar, regulating viral alternative RNA processing by internal side polyadenylation suppression and splicing of an adjacent upstream intron.VP1, VP2 and VP3 are expressed from the same transcript but have different start codons; however, all VPs share a common C-terminus sequence [32]. The N-terminal region of VP1 has a phospholipase A2 (pLA 2) domain which is conserved in all parvoviruses. pLA2 enzymatic activities help nuclear activity and endosome escape during the virus replication process [34]. The overexpression of VP2 protein is responsible for the formation of capsid-like structures. These structures show common characteristics similar to other parvoviruses but with uniqueness in surface topology [32].

The virus enters the host cell through clathrin-mediated endocytosis (Fig. 3B) [35]. It affects patients of different ages, but primarily children and less than 5 years [28, 30]. HBoV persists even after the infection and is fourth in position about its persistence. According to a study, its disease is often associated with a higher rate of other viral co-infection [36, 37]. HBoV1 infection may be asymptomatic or range from mild to severe conditions, such as cough, wheezing, fever, respiratory distress, hypoxia, pneumonia. These virus infections can also be associated with other viral infections. HBoV2 and HBoV4 were isolated from feces samples of non-polio acute flaccid paralysis, while HBoV3 was from the stool of children with diarrhea [31]. HBoV1 detected in the stool of gastroenteritis children suggests that this virus is both respiratory and enteric [38]. Quantitative PCR is a method for diagnostic studies of HBoV. In addition to this, other methods include IgG, IgM and enzyme-linked immunosorbent assay (ELISAs) and enzyme immunoassay using recombinant VP2 protein detection [30]. To date, no vaccine came into existence and also, from a medication perspective, this virus has not been affected. The symptomatic infection of this virus, which leads to pneumonia, can be treated with supplemental oxygen and wheezing by bronchodilators.

## Orthomyxoviridae

### Swine influenza virus (H1N1)

Swine flu (H1N1), caused by Influenza A virus (IAV) belonging to the *Orthomyxoviridae* family, is considered a significant contributor to respiratory infections. These viruses are with single-stranded, negative-sense segmented RNA and the segments are segregated into eight parts which help in genetic reassortment. These are spherical shaped enveloped viruses of 80–120 nm in size and share many similarities with paramyxoviruses causing respiratory infections (Fig. 3C). Animals serve as a natural reservoir of influenza, which causes epidemics. The disease is highly contagious and the symptoms in pigs are similar to humans and have been known as the model organism for influenza research. It has been reported that pigs are the “mixing vessels” for the viruses causing pandemic to the high susceptible avian, swine and humans. Infection in pigs is through indirect or direct contact with aerosols or asymptomatic pigs. The transmission of the swine flu virus takes place from pigs to pigs and pigs to humans [21]. Every year 3–5 million cases of severe illness and deaths are reported worldwide. The first influenza outbreak occurred in the eighteenth century in Russia and began spreading across the globe [39]. In the twentieth century, the Spanish flu (H1N1) pandemic appeared in 1918 and 20–40 million people died. Similarly, in 1957 Asian flu (H2N2), and the 1968 Hong-Kong flu (H3N2), caused 1 million deaths due to these pandemic outbreaks. In 2009, up to 575,000 people have died worldwide due to the H1N1 pandemic [39].

Orthomyxoviruses are divided into four different genera (influenza A, B, C and D) based on the antigenic nature of ribonucleoprotein present on the virus surface. These four types show significant differences related to their genetic organization, structure, possible hosts, epidemiology and clinical manifestations, being influenza A and B the most common human pathogens. Influenza viruses A (IAV) and B (IBV) cause seasonal epidemics in humans but influenza C (ICV) cause only mild disease. IAV is highly mutable and undergoes antigenic shift and drift but IBV shows only antigenic shift. ICV virus is highly stable in comparison with others. IAV is further classified into three subtypes based on the antigenic proteins, Hemagglutinin (HA) and Neuraminidase (NA), present on the viral surface. 18 subtypes of HA and 11 subtypes of NA being existed in animals. Among these three subtypes of HA (HA1, HA2 and HA3) and two of NA (NA1, NA2) are found in humans. Antigenic drift helps in infecting many species [40]. IBV and ICV are not divided into subtypes and are restricted only to humans, but two antigenically different strains of IBV (B/Victoria/2/1987-like and B/Yamagata/16/1988-like) circulate among the human population [41]. Influenza D virus (IDV), mainly found in cattle, is related to ICV. Influenza transmits through droplets generated in the air through sneezing, coughing or talking. It communicates over a distance of 3–6 feet. Contact transmission is possible by direct or indirect touching an infected person or objects touched by an infected person [42]. Influenza infections cause both respiratory disease symptoms [high-grade fever (38°), cough and sore throat, body ache, headache, and tiredness] and non-respiratory symptoms affecting other organs [43]. Symptoms of swine influenza in humans are distinguishable from influenza A viruses in humans. Mortality and severe illnesses have been reported due to severe pneumonia and super secondary bacterial infection in the lower respiratory tract [44].

Detection of influenza antigen can be carried out using nasopharyngeal aspirates and throat swabs by indirect fluorescent test (IFT) or ELISA tests. PCR-based serotyping can be performed for further subtyping [45]. Influenza infection can treat with the help of antiviral drugs, such as Amantadine, Rimantadine inhibiting M2 matrix protein ion channel. Neuraminidase inhibitors such as Oseltamivir, Zanamivir prevent viral release in a host cell. Influenza vaccines such as tube inactivated vaccines (TIV) and Live attenuated vaccines are currently available; however, these are not effective; hence there is a need to develop better treatment options [46].

### Avian influenza virus (H1N1)

Avian Influenza viruses are of two types based on clinical symptoms and disease severity. In 1999, Italy reported an epidemic of low pathogenicity avian influenza (LAPI) infection and after several mutations, a high pathogenic avian influenza (HPAI) emerged by the H7N1 subtype. High pathogenic avian influenza (HPAI) is also caused by H5 and H7 subtypes of the avian virus [47]. Influenza A virus was known to infect the avian species; however, through genetic drift and antigenic shift, the virus evolved to transmit to humans directly without any intermediate host [47, 48]. Like other types of influenza A virus, avian influenza belongs to the Orthomyxoviridae family. Although avian influenza infections in humans are rare, few subtypes have been reported using humans as reservoir hosts. Two subtypes of avian influenza virus (HPAI), H5N1 and H7N9, have been reported to be highly pathogenic in poultry and humans. From 1959 to 1990, the highly pathogenic avian influenza has been majorly circulating and are stable lineages reported in Europe, Asia, North America and Australia. The epidemics causing H5N1 virus lineage in Asia have originated in Guangdong province of the Peoples Republic of China. From the year 2003, H5N1 has been reported in countries, such as Japan, South Korea, Thailand, Vietnam, and mainland China. The circulation of H5N1showed that it has affected countries, such as Asia, Africa, and the Middle East. From 2005 to 2007, the case reports have increased gradually due to the spread of the virus clade 2.2. Another subtype HPAI (H7N9), in February 2013, in China first case has been reported to infect humans. From 2013, 1500 cases and several human deaths were reported [49–51]. A recent case report was confirmed on 5th January 2022 in England with H5N1 in an asymptomatic person who was known to rear the ducks at home[52].

Avian influenza in humans shows disease symptoms, such as fever, cough, pneumonia, and acute respiratory syndrome. The onset of infection has been reported from 4 to 10 days. Less common symptoms such as gastrointestinal and leukopenia, thrombocytopenia, and lymphopenia have also been diagnosed. In children, H5N1 isolated from Cerebrospinal fluid had affected the central nervous system with symptoms of diarrhea and coma. High viral load has been detected in diseased patients' upper respiratory tracts [49, 53, 54]. According to WHO, the clinical management of the avian human influenza virus can be done using antiviral drugs. Oseltamivir is the only primary antiviral drug treatment given at the early stages of infection. Antivirals, neuraminidase inhibitors, corticosteroids and immunomodulators have been regimed based on disease symptoms. The spread of disease can be controlled with self-isolation, and maintaining hygienic conditions [55].

## Paramyxoviridae

### Human respiratory syncytial virus (HRSV):

Respiratory Syncytial virus, recognized in 1959, was first recovered from the Chimpanzee, named Chimpanzee coryza agent, and later observed the formation of giant syncytia in host cells (tissue cultures); it was called as Respiratory Syncytial Virus (RSV). RSV infection was the most prevalent lower respiratory tract infection that was detected among a panel of common respiratory viruses [56]. This virus belongs to the *Mononegavirales* order, *Paramyxoviridae* family, *Pneumovirinae* subfamily, and genus *Orthopneumovirus.* RSV is one of the leading causes of death of infants of less than 1 year worldwide, second only to malaria. It is also clear that RSV is a prevalent and very serious respiratory pathogen in 65 years and above. A high percentage of infection has been observed among infants and children with high mortality rates. Although high infection rates were observed in young children, older adults with long term care facilities and immunocompromised individuals also have significant mortality rates [57].

RSV infections show a seasonal pattern of occurrence. A spike in infection is observed during winters in temperate regions while during summer and rainy seasons in tropical regions. In countries such as Europe, U.K, U.S, Spain, Italy, and Japan, there has been a steep increase in the deaths of infants among the age of less than 1 year. In Europe and the U.S, 90% of hospitalization cases were reported which is peak during January and February. In India, the RSV causing mild to acute Lower Respiratory Tract Illness (LRTI) ranges from 5% to 54% depending upon the age groups (infants and children less than 5 years) and seasonal variations [58]. Despite the substantial global burden of diseases, such as heart disease and chronic and pulmonary infections, acute lower respiratory tract infections contribute majorly to increasing death rates in several countries. Although lower respiratory tract infections are a leading cause of death in infants and young children, upper respiratory tract infections with mild to severe infections have also been observed in adults suffering from other pulmonary diseases or in immunocompromised. Immunity achieved after RSV infection is incomplete and reinfections are frequent. However, a high percentage of mortality measures disease severity in RSV infection [58].

RSV is non-influenza type enveloped virus (Fig. 3D). RSV contains an unsegmented RNA of 15.2 kb size. The viral genome has a specific gene sequence NS1‐NS2‐N-P‐M‐SH‐G‐F‐M2‐L in a 3′‐5′ direction with short noncoding terminal and intergenic regions separating each gene. The viral mRNA encodes eleven proteins, non-structural protein (NS1 and 2), Nucleoprotein (N), Phosphoprotein (P), Matrix (M), Short Hydrophobic (SH), Glycoprotein (G), Fusion (F), transcription processivity factor (M2‐1), (M2‐2), and large polymerase complex (L). The outer envelope contains transmembrane glycoproteins (G) for attachment to host cells, along with fusion protein (F) and short hydrophobic (SH) protein involved in the replication process (Fig. 3D). Variable G protein and F protein are known for antigenic determination, and based on this, the RSV is subdivided into two groups RSV-A and RSV-B. RSV A has 14 genotypes, while RSV B has 27 genotypes. The F protein is conserved among both groups but the G protein has homology sequence similarity of 53% and 67% with groups B and A, respectively [56]. F protein interacts with the host proteins, such as toll-like receptor-4 (TLR4), Intercellular Adhesion Molecule 1 (ICAM1), and Nucleolin. The main hRSV receptor for interaction is Nucleolin which makes cells permissive to the infection. Along with F protein, G protein aids in the mechanism of virus–host membrane attachments. The glycosylated G protein allows its binding with annexin 2 and heparin protein on the host cell surface. G protein's secreted form helps the virus escape opsonization and neutralization by G-specific antibodies. Another critical function is modulating the activity of host CD8+ T-cells by impairing the function of certain cytokines and chemokine by Chemokine receptor type 3 (CXC3) motif that interacts with host receptors [59]. The non-structural proteins are unessential accessory proteins but play specific functions in infected cells. NS1 and NS2 act as virulence factors that impair interferon's innate immune response in hosts. NS1 modulates the Janus Kinase/Signal Transducer and Activator (JAK/STAT) pathway by degrading the STAT proteins and also inhibits the phosphorylation of interferon regulatory factor-3 (IRF3). NS2 protein interferes with activation of IRF3 that interacts with retinoic acid inducible, there by inhibiting interferon innate response. Non-structural proteins are involved in immune response pathways but have also been reported to interact with mitochondrial and nuclear proteins, trafficking their vital functions. All other proteins have a role in replication processes [60, 61].

RSV has been the most infecting pathogen causing severe respiratory illness. Low levels of serum immunoglobin, nasal IgA and suppressed immune response make susceptible to RSV infection in elderly patients. Humoral immunity involving Th-1 cells, cell mediated immune response control the viral clearance. Plasma cells generating neutralizing antibodies may aid in viral clearance during infection. Pneumonia in infants is the major hallmark of RSV infection. Infection in the lower respiratory tract includes fever, cough, rhinorrhea, wheezing, intensive breathing and hypoxia, while severe conditions include grunting, intercostal retraction during breathing, nasal infiltration and respiratory distress. Neutrophil Intensive Inflammation occurs in both upper and lower respiratory tract infected infants and eosinophilia. Children with significant exacerbations such as asthma and chronic obstructive disease are reported in the case of severely infected with RSV [62, 63]. Viral isolation, enzyme immunoassay detection, plaque forming assay and immunofluorescence assay have been used widely for the detection. Other antibody neutralization tests and detection with multiplex RT-PCR are primarily used to detect single and co-infections. Treatment with attenuated vaccines and antivirals such as repurposed drugs has been put forward to cure these infections. Palivizumab and ribavirin are the only prophylactic drugs for RSV approved by the Food and Drug Administration (FDA) and symptomatic treatment and supportive care. Subunit vaccines have been reported to show potent efficacy against RSV infection in young children and adults. Purified F protein (PF1, 2, 3) subunit vaccines have been reported to be effectively safe and immunogenic for adults and young children. A recent report with phase 3 clinical trial showed the efficacy of recombinant F protein nanoparticles in a pregnant woman as safe and effective against RSV infection and in infants [64, 65].

### Human parainfluenza virus (HPIV):

The Human parainfluenza viruses (HPIV) were first obtained from children with lower respiratory tract infections and placed under the paramyxoviruses group (Parainfluenza viruses). HPIVs are important causes of upper respiratory tract illness (URTI) and lower respiratory tract illness (LRTI) and have evolved as the new classified group [66]. HPIV belongs to the family *Paramyxoviridae*, grouped into genera Respirovirus (HPIV 1 and 3) and Rubulavirus (HPIV 2 and 4) based on their genetic, antigenic serotype analysis. These viruses show genomic and structural resemblance with RSV viruses. The viral genome is organized in direction (3' N–P–C–MF–HN–L-5'). Unlike RSV, each HPIV has at least one non-structural protein and possesses Hemagglutinin Neuraminidase (HN) as the attachment/fusion protein along with the F protein. Tetrameric form of HN protein allows binding to host cell membranes via sialic acid receptors. Human Parainfluenza Viruses (HPIV2and 4) have two Phosphoproteins of 49–53 kDa size, while HPIV1and 3 of 83–90 kDa. The largest L protein, a polymerase enzyme, nucleoprotein (N) and nuclear phosphoprotein (NP) interact with viral RNA to form a replicating complex. HPIV1 encodes a non-structural protein C, whereas HPIV2 encodes a V (a protein involved in viral replication) and HPIV3 encodes protein D. Innate response, Tumor Necrosis Factor alpha (TNF), Interleukins (IL-1β, IL-6), Th1 and Th2 and cytokines are the host immune responses to HPIV infections. Glycoproteins are involved in the initiation of complement fixation pathways [62–64]. All HPIV serotypes occur at different intervals in a year. HPIV1 infection shows a biennial phase, whereas HPIV2 peaks in winters, HPIV3 and 4, peaks in mid summers and autumn. Each year, 5–8 million infections for LRI have been reported in the U.S, among which 12% cases are caused by HPIV (HPIV 1,2and 3). In the U.K (1975–1997), 70% of the cases were reported for HPIV3 infection. After RSV, it is the second most causing respiratory disease occurring in infants and young children. 17% of HPIV hospitalization cases were reported for children under the age of 5 years. Serotype surveillance shows that 60% of young children were infected with HPIV 3 and 80% infection by HPIV4. A study reported 62% croup cases in young children [67–69]. Both RSV and HPIV have a similar global disease burden in infants and young children but in adults, infection occurs with mild to severe manifestations. Therefore, epidemiological surveillance is a key factor in determining the circulating strains and type of prophylaxis during an outbreak. HPIV infection and its disease symptoms depend upon the serotype circulation. HPIV infects the upper respiratory tract (URT) and causes pneumonia and bronchitis, as observed in RSV. Croup is the significantly observed symptom of HPIV 2 in children, while HPIV 1 and 3 show bronchitis and pneumonia in infants and young children. Although very few cases of infection with HPIV 4 have been reported, a study reports a biphasic fever in 1-year-old infants. Furthermore, the upper respiratory tract infection accompanied by tracheobronchitis is also caused by HPIV4 infections [69, 70]. Formalin inactivated HPIV vaccines were failed for treatment in young children. Live attenuated HPIV-3 vaccine entered in phase 2 clinical trial for infants and was found to be safe, but the developed antibodies were not cross reacted with HPIV3. No licensed vaccine against HPIV has been developed, and some are still in clinical trials [71].

### Human metapneumovirus (HMPV)

Human metapneumovirus **(**HMPV) is an emerging virus causing both upper and lower respiratory tract infections. Like RSV, it belongs to the order *Mononegavirales*, family *Paramyxoviridae* and genus *Metapneumovirus*, first discovered by van den Hoogen et al*.* in The Netherlands in 2001 from children samples with acute respiratory illness [72]. It had been circulating undetected for a long time in the human population worldwide [73, 74]. The metapneumoviruses are enveloped, non-segmented, negative-sense, single-stranded RNA with an approximate size of 13 kb. They comprise a genus of two species: avian metapneumovirus and HMPV. The genome encodes nine proteins viz nucleoprotein (N), phosphoprotein (P), matrix protein (M), fusion protein (F), matrix-2 proteins (M2-1 and M2-2), small hydrophobic (SH) protein, glycoprotein (G), and large (L) polymerase. These proteins play essential roles from fusion to viral replication and translation [75, 76]. F protein binds to the cell surface integrin and is further aided by viral G, SH and M2-2 proteins in entering host cells (Fig. 3D). The ultimate consequence is the influx of immune cells due to inflammation and erosion of respiratory endothelial cells [77]. This virus has two genotypes A and B based on the membrane glycoproteins F and G which are further divided into two clades each, i.e., A1, A2, B1 and B2 and two sub-clades A2a and A2b. Co-circulation of the genotypes or sub-clades has been reported, but the strain’s predominance can vary from year to year [78].

HMPV has a seasonal distribution similar to that of other respiratory viruses and tends to peak in later months compared with RSV and influenza. This virus is a leading cause of acute respiratory infections in children, immunocompromised and elderly patients. Transmission occurs by direct contact with an infected person or objects. Fever, sore throat, cough, wheezing, breathing difficulty, hypoxia, bronchitis, and pneumonia are the major symptoms associated with the HMPV infection [74]. Seroepidemiology studies have shown that most children worldwide are infected with HMPV by 5 years of age. Young children under the age of 5, including infants, chronically diseased persons mostly with asthma, cystic fibrosis or lymphoma, immunocompromised persons and those above 60 years of age are more prone to infection by HMPV. Throughout an individual's life, reinfection occurs once infected by HMPV [77]. It causes 6–40% of acute respiratory illnesses globally with 3–5 days of incubation. HMPV tends to cause mild infection in other age groups except in the elderly, and it causes severe disease, including pneumonia and bronchitis, with as high as 50% of mortality and morbidity among 60% of those hospitalized [79]. The annual rate of HMPV infection is estimated to be 55 per thousand in the case of children with a 1 per thousand rates of hospitalization, whereas in adults, a 3% rate of infection is observed compared to children, excluding complicated cases [80, 81]. Studies reported that co-infection with other viruses and bacteria exacerbates symptoms and disease but not the severity of the disease [73]. The symptoms range from mild (cough, rhinorrhea, and fever) to more severe (bronchiolitis and pneumonia) and are similar to other respiratory viral infections.

The diagnosis of HMPV depends upon nucleic acid amplification and subsequent reverse transcriptase-PCR targeting the conserved gene of the virus, commercial multiplex assays and real-time PCR [82–84]. Virus culture remains obsolete due to the long propagation time, the requirement of exogenous factors and the robust cytopathic effect [73]. No licensed antiviral drug is available to treat the HMPV infection, although Ribavarin and immunoglobulin have been investigated for treatment [85]. Both treatments have efficacy in in vitro studies and mice, but no recommendation for human use has been given [86]. Research on the development of vaccines against HMPV is still underway globally. Many vaccine candidates have been put forward for trial after experimentation on animal models. Some of which include live attenuated vaccine, recombinant vaccine lacking the G, M2-1, M2-2, Short Hydrophobic (SH) proteins, mutated strains in the polymerase and Arginine, Glycine and Aspartate motif (RGD motif) of Fusion protein (F), heat-killed or formalin-inactivated virus and lastly vectored vaccine. Increased antibody levels were found in response to these types of vaccines with no effect on the viral replication cycle. Similar to formalin-inactivated RSV (FI-RSV) and FI-HPIV, the formalin-inactivated HMPV vaccine proved to increase the disease severity following infection by some other viruses. This phenomenon led to a rise in the level of cytokines and ultimately lung inflammation when such candidates were tested in animal models, such as rats, hamsters, etc. This proved them to be insufficient for use as a vaccine candidate. Besides the above, subunit vaccines which use viral protein have shown good amounts of immunity, but studies also reported that the immunity induced by such vaccines tends to decrease over time and may require later immunization. Peptide vaccines based on cytotoxic T lymphocyte (CTL) epitopes and Multi-epitope peptides (MEP) have been developed for testing. These types of peptides reduce viral load and increase the T and B cell response, indicating strong humoral and cell mediated immunity [87].

## Picornaviridae

### Human enteroviruses (EV-D68)

Human enteroviruses (EVs) comprise a large group of non-polio viruses, causing respiratory illnesses besides hand, foot and mouth disease, encephalitis, etc. It belongs to the family *Picornaviridae*, genus *Enterovirus* and species *Enterovirus D* (EV-D68). Since its first isolation from California in 1962, it has been distributed worldwide, causing mini outbreaks mainly in the US, Europe and Asian countries. From 1970 to 2005, only irregular outbreaks were reported by the US NESS, but from 2005 frequent outbreaks were reported with a worldwide increase in the numbers till 2014 [88]. In the US, major public concern was raised on the EV-D68 infection along with European countries, such as Norway and France, whereas in The Netherlands, the virus was found to be circulating for four consecutive years until it was detected in 2014 [89, 90]. Later in 2016, cases of infection were reported from the majority of the other European countries, such as Portugal, Sweden, the UK, and Italy, of which young children were affected in association with acute flaccid myelitis (AFM) [57, 91]. EV-D68 belongs to one of the four groups of enterovirus genus that infect humans. It has a 7.6 kb ( +) sense ssRNA as genome with an ORF coding for four structural (VP1 to VP4) and seven non-structural proteins (2A to 2C and 3A to 3D) flanked by 5′ and 3′ Untranslated region (3′UTRs) either side with a 3’ poly adenylation (poly(A)) tail [90, 92]. VP1 viral protein helps to detect different serotypes due to the presence of a serotype-specific loop and proteins 2C and 3C have been targeted for antiviral therapy because of their role in the virus infection cycle. Clade A, B, C and D are the four subtypes of EV-D68. Clades A and B are further sub-classified as A1, A2 and B1 to B5, but after the 2014 outbreaks, two new clades were found to be circulating, namely, B3 and D [93]. Different clades or sub-clades have been co-circulating as in the US, clades A1, B1, B4 and B5; European countries A1, A2, D, B1 and B2 and in Asian countries B, C, D and B2 since 2014 [94]. Changes in the circulation pattern of the clades have been attributed to recombination and mutation frequency accompanied by the degree of polymorphism in humans of a particular geographical region and immune responses.

Infection occurs in all age groups from 4 years, but the fatality rate is observed in the case of immunocompromised patients. Transmission occurs through droplets of infected people or infected objects and the virus has an incubation period of 1–2 weeks. The symptoms of enterovirus EV-D68 include fever with cough, sore throat, rhinorrhea, bronchitis and exacerbation of asthma in young patients, including lower and upper respiratory tracts. Studies have reported an association between EV-D68 infection and acute flaccid myelitis, but a more in-depth analysis is required to reveal the underlying mechanism [91]. EV-D68 infection is reported to suppress the innate immune response by eliciting a replication cycle due to less production of interferons and manipulating the progression of the cell cycle by decreasing the cyclins and Cyclin Dependent Kinases levels and inducing the cells to enter the G0/G1 phase for more viral production [93, 95, 96].

The diagnosis of EV-D68 infection is through multiplex assays, RT-PCR and duplex assay for D68 positive and non-D68 cases. Although 16 drugs have been tested in the US as an antiviral treatment for EV-D68 infection, no approved drug or vaccine is available [88]. Some drugs such as rupintrivir (protease inhibitor), enviroxime, V-7074, DAS-181, capsid binder such as pleconaril, vepandivir, pocavir have shown inhibition; there seem to be limitations[26, 97]. In addition, monoclonal antibodies, namely, 15C5 and 11G1, have also decreased EV-D68 infection through their neutralizing mechanism and binding to viral proteins [98]. Proper understanding of the host–viral interaction, the evolution of the virus, and periodic surveillance is required to manage outbreaks. Supportive care remains the sole strategy for treatment unless requiring ventilator support and corticosteroid administration in severe conditions [92, 99].

### Rhinovirus A, B and C

Human Rhinovirus (HRV), the leading cause of frequent `common cold’, is characterized clinically by symptoms, such as nasal congestion, headache and sore throat. This virus was first identified in 1956 in patients with upper respiratory tract infections. It is recorded as the second leading causative agent of the upper respiratory tract (URT) and bronchitis in children, the elderly and immunosuppressed people [100–103]. HRV causes respiratory infections in billions of people annually and accounts for 52–76% of infections [104, 105]. Globally, HRV is circulating in the human population, even in remote areas, such as Bushmen tribes from the Kalahari Desert and Alaskan. It has been a frequent cause of the common cold in the US and Western Europe. It accounts for 80% of autumn colds in the US and tropical areas. The indoor transmission of rhinovirus is favored by humidity during the rainy season. These viruses are the most common cause of self-limited upper respiratory tract infections in immunocompetent adults. They could be responsible for more than 80% of common colds in the autumn and spring seasons [104].

HRV is a non-enveloped spherical virus of the family *Picornaviridae* and genus *Enterovirus* [106]. The Rhino viral genome is a single stranded 7.2 kb positive-sense RNA that translates into a single polyprotein and is cleaved by viral proteases into several structural and non-structural proteins. The icosahedral capsid comprising three cell surface capsid proteins VP1, 2 and 3 and another capsid protein VP4 will be present below the capsid. Various non-structural proteins such as 2A, 2B, 2C, 3A, 3B, 3C and 3D are produced after proteolytic cleavage of a single polyprotein [107–109]. There are more than 160 Rhinovirus strains which are classified into 3 species of Rhinovirus, i.e., RV-A (~ 80serotypes), RV-B [32 serotypes] and RV-C (~ 60 serotypes) based on capsid properties and conserved sequences[103, 110, 111]. The RV-A and B serotypes are further classified into minor and major group serotypes based on their cell surface receptor’s specificity toward intercellular adhesion molecule 1 (ICAM-1). Sixty-eight of the RV-A serotypes and all RV-B serotypes are included in the major receptor group. 12 RV-A serotypes have specificity to the low density lipoprotein (LDL) receptor family and belong to the minor receptor group [107, 109]. RV-C serotypes bind to cadherin receptor CDHR3, which is involved in susceptibility to childhood asthma [111]. Rhinovirus infection generally initiates after self-inoculation of the virus into the nose and eyes [106]. The rhinovirus attaches to epithelial cell receptors of an adenoid area of the nose, followed by conformational changes in the capsid protein and leading to the release of viral RNA (Fig. 3E) [112]. The viral RNA will be synthesized and further translation will start using the Internal Ribosomal Entry Site (IRES) mechanism. The mature virions are released from epithelial cells after 8–10 h by cell lysis (Fig. 3E). Cells of innate and adaptive immune systems involving macrophages, neutrophils, eosinophils, T-cells and B-cells elicit responses against HRV. Serotype specific antibodies such as IgM, IgG and IgA develop in case of severe infections within 7–21 days. However, evidence indicates that rhinoviruses manipulate the immune system [113]. Co-infection of HRV with other respiratory viruses such as HRSV is evidenced, but the severity of HRV exhibited symptoms is still questionable [114, 115].

Due to limited virus culture facilities, the diagnosis of HRV was difficult. Still, after advancements in nucleic acid amplification techniques, RT-PCR is being used to detect HRV in lower respiratory tract infections. Ohio Hela cells were first used to isolate human rhinovirus, which can be used to detect respiratory secretions. Primate cell lines facilitate rhinovirus propagation in cell culture, but HeLa and embryonic fibroblast cells of human origin can also be used. The optimum temperature conditions for growth are 33–35℃ for 10–14 days in culture. RT-PCR-based assays that detect conserved 5` UTR can be used for serotyping [116]. Several antiviral agents for Rhinovirus treatment are in trial mode, but none provide viral infection resistance. The polyvalent inactivated HRV vaccine could reduce severe illness in asthma and Chronic Obstructive Pulmonary Disease (COPD) patients [117].

## Coronaviridae

### Human coronavirus

Human coronavirus is the causative agent of respiratory tract infections and is the second most common cause of colds, with an average 15% of total acute respiratory tract infections in the U.S, varying from 1% to 35% annually. Severe infections of the lower respiratory tract-like pneumonia have also been documented in elderly, young and immunosuppressed people. The WHO estimated that out of 450 million cases of pneumonia, 200 million are community-acquired coronavirus infections [118]. The coronaviruses (CoVs) members are the largest group of viruses belonging to the order *Nidovirales*, including the *Coronaviridae, Arteriviridae, and Roniviridae* families. The family coronaviridae consists of two subfamilies *Coronavirinae and Torovirinae.* The *Coronavirinae* are subdivided into four groups: alpha, beta, gamma, and delta coronaviruses, based on sequence comparisons of entire viral genomes [119]. The viruses were initially sorted into these groups based on serology but are now divided by phylogenetic clustering. These CoVs can infect various hosts, including avians, swine and humans. There were six human coronaviruses (HCoVs) identified; HCoV-OC43, HCoV-229E, HCoV-HKU1, HCoV-NL63 and severe acute respiratory syndrome-coronavirus-CoV1 (SARS-CoV1) and Middle East Respiratory Syndrome coronavirus (MERS-CoV) before the SARS-CoV2 identifed as the seventh human coronavirus. These HCoVs are classified either in the Alpha- or Beta coronavirus genera as two Alpha coronaviruses (HCoV-229E and HCoV-NL63), and five Beta coronaviruses (HCoV-HKU1, SARS-CoV1, MERS-CoV, HCoV-OC43 and SARS-CoV-2). Among the total seven HCoVs, four (HCoV-229E, HCoV-NL63, HCoV-OC43 and HCoV-HKU1) are globally circulated in the human population. Furthermore, the seven CoVs which are known to cause human diseases are divided into low and high pathogenic CoVs [120, 121]. Four coronaviruses (HCoV 229E, NL63, OC43, and HKU1), are known as non-severe acute respiratory syndrome-like CoVs. They cause mild diseases and are globally endemic. Over the past two decades, SARS-CoV1, MERS-CoV and SARS-CoV-2 have emerged as novel zoonotic CoVs, highly pathogenic, which cause lethal human disease. These SARS CoVs exhibit different mechanisms to evade the host immune response and hence cause severe disease [122]. The SARS-CoV-1 was discovered in November 2002, the MERS-CoV in June 2012 and SARS-CoV-2 was found in 2019 [123]. Initially, SARS-CoV-2 was named 2019-nCoV when it was identified in December 2019 after sequencing clinical samples from a cluster of patients with pneumonia in Wuhan, China. The disease caused by SARS-CoV2 is named as Coronavirus Diseases-2019 (COVID-19). SARS-CoV2 appears to be a highly human-to-human transmissible pathogen and causes a wide spectrum of clinical manifestations [124]. Unlike other respiratory diseases that have a quadratic ("U"-shaped) lethality curve (killing infants and elderly but sparing adults), SARS**-**CoV-2 has a lethality that continuously rises with age (sparing children but mainly killing the elderly). OC43 causes common colds less frequently and is seasonable, mainly in winters and spring. HCoV229E and OC43 cause winter outbreaks in 2–4 year intervals in temperate regions. There is no evidence of HCoV229E and OC43 infections in tropical countries. HCoV-NL63 is mainly responsible for LRTI in 45% of infants, while HKU1 can cause LRTI in adults also. HCoV-229E was the most common cause of mild common cold worldwide, discovered in 1966 in Hipposideros bats from Ghana. HCoV-NL63 was first isolated in 2004 from infected children suffering from pneumonia and bronchiolitis, of which approximately 9.3% of HCoV–NL63 [125, 126]. HCoV-OC43 was identified in 1967 from National Institute of Health (NIH), USA, employees infected with acute upper respiratory tract illness. It contributes to a total of 11% of coronavirus infections. HKU1 was discovered in 2004 from an older man suffering from chronic obstructive airway disease in Hong Kong [127].

Being the coronaviruses as animal and human pathogens, animals serve as a reservoir and infect humans when they come in close contact with individuals. Symptoms of coronavirus infection are sore throat, nasal congestion, fever, bronchiolitis and pneumonia. HCoV infections have also been associated with asthma and wheezing. Notably, SARS-CoV-2 was closely related (with 87–92% identity) to two bat-derived severe acute respiratory syndrome (SARS)-like coronaviruses, bat-SL-CoVZC45 and bat-SL-CoVZXC21, collected in 2018 in Zhoushan, eastern China, but were more distant from SARS-CoV-1 (about 79%) and MERS-CoV (about 50%). Coronaviruses are large, enveloped with petal shaped spikes at their outer surface, making a crown-like appearance, and hence the name ‘coronavirus’ has been attributed (Fig. 3F) [128]. The genome is a positive sense single stranded 27–32 kb RNA and the helical nucleocapsid is covered by a viral envelope. This positive sense viral RNA acts as mRNA and the virus RNA synthesis occurs in the cytoplasm, which requires a negative sense RNA as an intermediate (Fig. 3F). The RNA is the largest known viral RNA and codes for a large polyprotein. Two UTRs flank the viral RNA (5′ and 3′ UTRs). The 5′ end is capped and the 3′ end is poly adenylated (poly A tail). Two-thirds of the viral genome encodes for two overlapping ORFs: ORF1a and ORF1ab, which encode 16 non-structural proteins. One-third part encodes for four structural proteins for which the single ORF translates into a polyprotein which further cleaves into four structural proteins S (Spike), M (membrane), E (Envelope) and Hemagglutinin Esterase (HE) in some viruses. S protein contains cell receptor ligand for T cell binding. M protein interacts with N (nucleocapsid) protein embedded in an envelope. The viral encoded protease cleaves polyprotein into non-structural proteins, such as RNA-dependent RNA polymerase (RdRp) and ATPase Helicase [129].

### Severe acute respiratory syndrome (SARS-CoV-1)

At the beginning of the twenty-first century, SARS-CoV-1 was identified in humans and its origin was found to be in bats [130]. The first case of SARS was reported in Hanoi, Vietnam, by Carlo Urbani, a WHO scientist [131]. Its first outbreak was reported in the prince Wales hospital in early March 2003 and next few months it got spread to North America, South America, Europe and was finally recorded as a global outbreak. WHO estimated 8,098 people were infected with this coronavirus and approximately 774 people died during the 2003 outbreak [132, 133]. After the incubation period of 2–7 days, the fever begins, followed by shortness of breath and unproductive dry cough [134]. The disease symptoms include fever, shortness of breath, myalgia, malaise, diarrhea, headache, and shivering (rigors). Transmission of SARS-CoV occurs from person to person by virus excretion in respiratory secretions and stool [135]. This enveloped SARS-CoV has a similar genome to the human coronavirus, a positive sense single-stranded RNA. The RNA genome consists of approximately 29 kb size and consists of nine open reading frames that encode structural and non-structural proteins [136]. The structural proteins are spike(S), envelope (E), membrane (M), and nucleocapsid protein (N). There are 16 non-structural proteins resulting from the polyprotein's cleavage by virally encoded protease. The RNA genome has a cap at 5′ UTR and 3′ UTR is with a polyadenylation tract [134, 137]. Viral entry into the host cell occurs with the interaction of human cell receptor angiotensin converting enzyme 2 (ACE2) with glycosylated spike protein of the virus [138]. In internalization, conformation change in S protein leads to membrane fusion and viral RNA is released into the host cell and translates into viral polyprotein (Fig. 3F) [139]. The spike protein of coronavirus is cleaved into two subunits by proteases, such as trypsin, factor Xa and cathepsin L. The S1 subunits have a receptor binding domain (RBD) and the S2 subunit helps bind with the host cell receptor and this protein plays a key role in viral infection and pathogenesis [139]. The RBD of S protein has reached the preclinical studies in developing a vaccine candidate [140]. The envelope protein consists of a transmembrane domain incorporated into the virion lipid envelope along with membrane (M) and spike protein (S), forms pentameric α-helical bundles, and has cation-selective channel activity. This activity of E protein can be reduced by hexamethylene amiloride (HMA) [141]. The transmembrane glycoprotein M has a triple domain that forms one-third of the entire protein and plays an important role in viral specific humoral response and can neutralize antibodies in virus infected patients effectively [139]. The nucleocapsid protein (N) associates with replicase transcriptase complexes (RTCs) which are essential for the infectivity of viral RNA. The N protein is essential for incorporating genetic material into coronavirus particles and forming a ribonucleoprotein complex. Its N- and C-terminal domains fold independently and have binding properties with the genomic RNA of the virus. A study showed that the N protein of SARS-CoV has self-interacting properties to form an oligomer [142]. Non-structural protein 1 (nsp1) inhibits gene expression in association with host 40S ribosomal subunit by blocking mRNA translation and also induces endonucleolytic mRNA cleavage in 5’UTR, which results in acceleration of 5′–3′ exonucleolytic mRNA decay pathway [143].

Diagnosis of a SARS-CoV-1 infection was established by reverse transcription-polymerase chain reaction (RT-PCR), culturing of the virus, or serological assays. Monkey kidney cell line was first used to isolate SARS-CoV-1. The virus's incubation in Vero E6 cell monolayer for a few days produces cytopathic effects. It has been observed that pooled throat, nasal swabs, rectal swabs, nasopharyngeal swab specimens are being used for the detection by RT-PCR [144]. An inactivated SARS vaccine developed by Sinovac Biotech was under phase 1 clinical trials [145]. In vitro cell culture revealed that chloroquine interferes with the terminal glycosylation of ACE2, elevating the endosomal pH, leading to the inhibition of viral infection [146]. The risk factor for SARS is those who had direct contact with an infected person and hence avoiding such interaction helps control the disease.

### Severe Acute Respiratory Syndrome Coronavirus 2 (SARS CoV2)

**Fig. 4:**
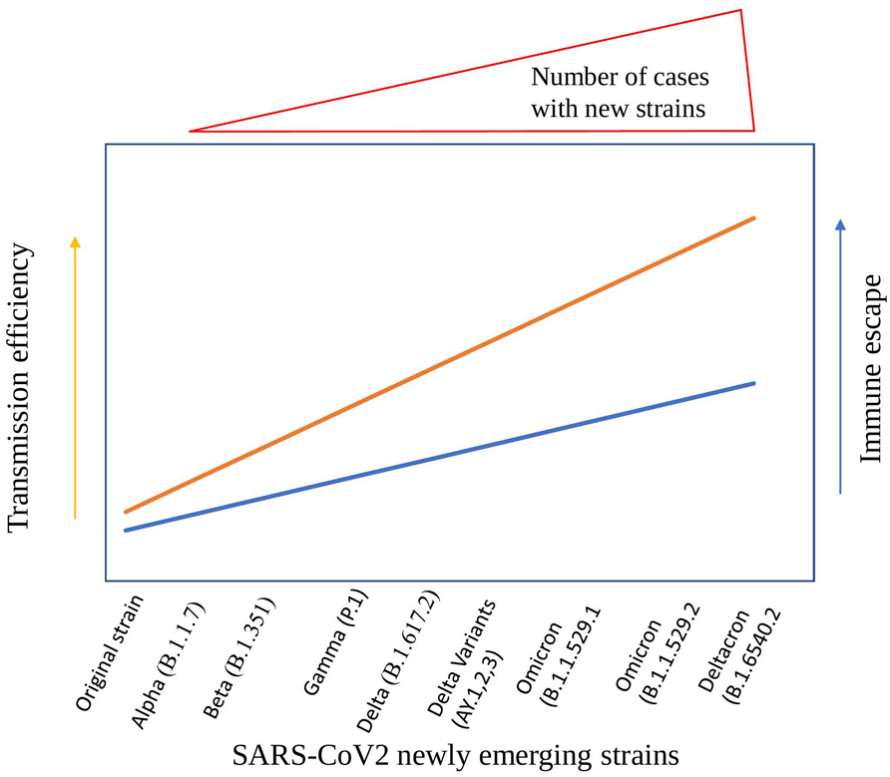
Predicted diagrammatic representation showing the emergence of the new SARS-CoV-2 strains and their possible increased efficiency in transmission and immune escape

The coronaviruses viz SARS-CoV-1, MERS-CoV and SARS-CoV-2 are at the top of the list of viruses (adeno, influenza and parainfluenza, respiratory syncytial virus, picornaviruses etc.) causing severe respiratory infections. SARS-CoV-2, the disease caused known as COVID-19, was reported in the year 2019, reached a world pandemic at the fastest rate in the history of human virus outbreaks. These virus infections spread to more than 228 countries infecting nearly 621 million people with a death toll of above 6.5 million as of October 2022. The devastation is continuing. Novel coronavirus, initially named by International Committee on Taxonomy of Viruses (ICTV) or SARS-CoV-2 as named by the WHO, causes severe acute respiratory syndrome. The new SARS-CoV-2 was first reported in December 2019 in a city of China, Wuhan, in Hubei Province and spread across the countries by the end of January 2020. WHO declared the outbreak as Global Public Health Emergency and the disease a pandemic [147]. These virus infections have been recorded in nearly 228 countries across the globe, with majorly contributing countries, such as the China, U.S.A, Brazil, India, Russia, Italy, the U.K, etc. [148, 149]. Despite several precautions, there was a daily increase in infections that simultaneously spread to almost all countries. Mortality from one country to another has differed depending on the number of people tested, healthcare delivery, population demographics and factual reporting. There is no virus in the history of viruses that caused as much as fast transmission and mortality. SARS-CoV-2 belongs to the *Coronaviridae* virus family, the SARS-CoV-1 and MERS-CoV also belong to, this virus exhibit significant divergence. Although the percent of mortality of SARS-CoV-2 (3–4%) is lesser than SARS-CoV-1(15%) and MERS-CoV (34%), the SARS-CoV-2 caused incomparable deaths in total. This may be due to specific point mutations in spike protein, which might bring efficient transmission and immune escape. In this direction, the SARS-CoV-2 variants viz Alpha (B.1.1.7), Beta (B.1.351), Gamma (P.1) and Delta (B.1.617.2), its variants (AY 1, 2, 3) Omicron and Deltacron are emerged (Fig. 4) [150]. These variants show the amino acid mutations mostly in the receptor binding domain (RBD) of spike protein. These variants are reported to be high virulent and cause up to 4^th^ phase of infections across the globe. In addition, delayed action of innate immunity might aid the greater number of severe infections and, hence, increased deaths (Fig. 4). There was a peak in number of cases across the globe, immediately after the emergence of these variants. The other uniqueness of this virus is the mortality rate caused among the aged and the people with prior health complexities. This characteristic was not recorded with any of the other viruses until now.

SARS-CoV-2 is a phylogenetically related new lineage of the genus beta coronavirus. It has 87–92% identity to bat coronaviruses (bat-SL-CoVZC45 and bat-SL-CoVZXC21) and is genetically distinct from SARS-CoV-1 and MERS-CoV by 79% and 50% identity, respectively. The virus characteristic and the pathogenicity studies obtained during SARS-CoV-1 and MERS-CoV were considered templates for understanding the SARS-CoV-2. Based on the homology modelling method, such as SARS-CoV-1, SARS-CoV-2 also binds to the same receptor ACE2 domain with variable amino acid residues (Fig. 3F). SARS-CoV-2 genome is similar to the beta coronaviruses with outer envelope protein bearing a structural spike protein. This protein helps in viral attachment and entry into host cells by endocytosis through the ACE-2 receptor (Fig. 3F) [124, 140]. This virus infects lung alveolar cells and gastrointestinal epithelial cells. Recent findings suggest that viral RNA detection is negative in some patients. There is viral clearance from the respiratory tract, but the infection persists in the gastrointestinal tract and may cause fecal–oral transmission [141]. According to WHO, the virus is contagious through person-to-person contact, infected person aerosols, sneezing, cough and air droplets. A study of the virus stability on surfaces and aerosols indicates the infectious nature of the virus. SARS-CoV-2 can be infectious in aerosols for hours (1.1 h), on solid surfaces such as stainless steel (5.6 h) and plastic (6.8 h) and for up to days on other surfaces [148, 151, 152]. Transmission of virus depends upon the Reproduction number (R_0_). As per the WHO estimates, the R_0_ of SARS CoV2 is 3.2 (median range 2.79), more than the reference standard (1.4–2.5). It is almost similar to the SARS-CoV-1 [2–5], although the SARS-CoV-2 transmission is more than SARS-CoV-1 [153]. A recent study suggests that pre-symptomatic (period of onset of symptoms) individuals may cause infection within 1–3 days while manifesting any symptoms. To estimate the COVID-19 spread, identifying nearly 40–50% of pre-symptomatic and asymptomatic individuals became a challenge. After the virus exposure, the virus incubates for at least 4–5 days and the patients may show symptoms after 11–12 days of infection [154]. Thus, a 14-day isolation period for patients showing mild general signs has been recommended.

The COVID-19 disease manifests common symptoms, such as fever, diarrhea, nausea, cough, dyspnoea, fatigue, myalgia, pneumonia, upper respiratory tract infections and organ dysfunctioning [e.g., shock, acute respiratory distress syndrome (ARDS), acute cardiac injury, and acute kidney failure]. In some patients, mostly adults were observed with comorbidities to respiratory failure due to severe alveolar damage. Some patients represented normal or lower white blood cell counts, lymphopenia (reduced T-cells, B-cells, Natural Killer cells, monocytes, eosinophil and basophils), thrombocytopenia, increased C-reactive protein and pro-inflammatory cytokines [152]. Among children, gastrointestinal, pharyngeal erythema and asymptomatic infections were observed. The prominent signs of pneumonia are decreased oxygen saturation, blood gas deviations, visible changes in lungs having alveolar exudates and interlobular involvement [155]. Patients infected with virus infection show higher levels of cytotoxic lymphocyte markers (NK cells and CD8+ T cells). During the convalescent phase, exhaustion markers (NKG2A, CD4+ T cells, B cells and NK cells) on lymphocytes get back to normal levels [156]. Several viruses, including coronaviruses, apply different strategies to escape from the host defense mechanism [122, 157, 158]. In this direction, SARS-CoV2 appears to possess a few unique characteristics which aid in fast-spreading and cause severe illness. In general, the genome size of the DNA viruses will be larger (100–200 kb) and hence some part of the genome will be dedicated to evading the host immune system. The genome size of the RNA viruses will be lesser [7–12 kb], but these viruses evade the immune system by undergoing frequent mutations. In this context, the genome size of the coronaviruses is larger (~ 30 kb). Hence, these viruses may apply two mechanisms in evading the host immune system, which is advantageous for the virus in fast spreading and in causing severe illness. The two mechanisms are 1. the ability to dedicate some portion of the genome and 2. undergo mutations (being an RNA virus). In addition, the ligand (spike)–receptor (ACE2) interaction appears to be irreversible (in the case of many viruses, this step is reversible) in the case of SARS-CoV-2 virus and hence leads to the nearly 100% infection in exposed cases [159–161]. These unique features gained by this virus are due to the genetic mutations and the additional mutations are already posing an increased threat.

Detection of SARS-CoV-2 has been recommended using the Bio-Safety Level-2 (BSL-2) facility. However, some laboratories have continued their procedures in BSL-3 safety cabinets [162]. Collecting the proper specimen at the right stage, right time from the right anatomic site is essential for coronaviruses' prompt and accurate molecular diagnosis. SARS-CoV-2 can be detected from samples collected from a nasopharyngeal swab or an oral pharyngeal swab. The high viral load persists in both upper and lower respiratory tracts during the onset of symptoms, allowing early infection screening. The most common detection method used based on immune responses are the rapid immunoassay that detects SARS-CoV-2 antibodies and antigens. Immunoassay includes lateral flow assay for detecting IgM and IgG antibodies against SARS-CoV-2. The rapid test kits for easy screening are accessible in many government and private laboratories: the SARS-CoV-2 antibody test, COVID-19 IgM IgG Rapid Test, etc. Molecular methods include real-time RT-PCR and Deep sequencing methods etc. Real-time RT-PCR allows the rapid diagnosis of the SARS-CoV-2 genome amplification with minimum false-positive results. However, there are different molecular targets for amplification, WHO recommends the Envelope (E gene) and RdRp gene for initial screening in real time RT-PCR assay. Unlike the gold standard test for viral detection, virus culture is not used as the method for SARS-CoV-2 detection. Deep and next-generation sequencing methods have been proposed to analyze the mutations but are not the true diagnosing methods [133, 163, 164]. In a recent study, contradicting these rapid detection tests (RT-PCR or nucleic acid test for COVID-19), the Computed Tomography (CT) scan imaging reported higher sensitivity which avoids misinterpretation of the clinical symptoms [165].

Based on clinical symptoms, repurposed or off-label drugs have been used to treat infection in an emergency conditions. These include Fivipiravir, Hydroxychloroquine, Remdesivir, a combination of Lopinavir and Ritonavir, Tocilizumab, Itolizumab, Dexamethasone, Methylprednisolone, Low molecular weight Heparin, Azithromycin, Ivermectin, Convalescent Plasma Therapy (approved as off label therapy), etc. Social distancing, isolation and quarantine remain the sole way of controlling the virus spread and WHO announced the possibility of many outbreaks shortly if these implementations are violated. WHO and the coalition of epidemic preparedness innovations (CEPI) developed a task force for COVID-19 vaccine development that takes care of the financing and manufacturing of vaccine [166]. The developed vaccine candidates include RNA, DNA vaccines, inactivated vaccines, protein subunits, non-replicating viral vectors, and replicating viral vectors [150, 167, 168]. Some potential challenges in COVID-19 vaccine development were put on as follows: 1.The long term antibody response 2. Decrease in the protective immunity even after a vaccine shot called antibody waning. 3. Antibody-dependent enhancement (ADE) is a phenomenon well-known in dengue cases, where immune cells cause adverse effects by enhancing replicating virus count. 4. Low immune response in persons over 60 years of age than young and adults and 5. Providing long-term immunity and sufficient immune response is called sterilizing immunity. However, certain pitfalls are still left in the developed vaccines concerning safety and efficacy. The need for the vaccine was in an emergency due to the rapid increase in active cases. This led to the development of the vaccine at a very fast rate within 12–18 months, bypassing the long 10 years of traditional vaccine development protocol that were being followed. Despite all the challenges, researchers, vaccine developers, funding agencies, and different governmental organizations worldwide, through their continuous support and mutual understanding, continued to find the best possible vaccine and made it available for mass vaccination by early 2021.

## Conclusions

Infection by any kind of viruses and bacteria spreads at a faster pace till the preventive measures are not implemented. Effective preventive measures and timely control measures for any disease outbreak are the first step of defense action toward the containment of infection. Viruses causing the respiratory infections show close but distinct characteristics. Hence, this review begins with the analysis for the taxonomic status of the viruses in the study and drawn the following conclusions. The review has discussed the viral respiratory infection mechanism, epidemiology, pathogenesis, disease symptoms, and related drugs and vaccines. The analysis suggested that viruses of six different virus families target the respiratory tract (Additional file 1: Table S1) [6–10]. The genome of the majority of these viruses is single stranded RNA. Being the RNA more prone to mutations, the emergence of variants for these viruses is highly possible. All the viruses are found to enter the respiratory tract by inhalation of the virus contaminated air or by physical contact. The members of the families adeno, parvo and orthomyxoviridae show similarities in their life cycle (Fig. 3A, B and C). The disassembly of these virus particle takes place in the nucleus. The life cycle of the viruses of the paramyxo, picorna and coronaviridae families show similarities (Fig. 3D, E and F). Entire life cycle of these viruses is restricted to the cytoplasm. In spite of many common characteristics of SARS-CoV-2 (causing COVID-19 disease) with other respiratory viruses, exhibit uniqueness in transmission, disease severity and death. Hence, in-depth analysis is required to decipher the uniqueness of SARS-CoV-2. Even though the non-COVID-19 respiratory viruses mostly cause mild-to-moderate disease, they may also be responsible for severe disease with need of ICU admission for supportive care. The incubation period of these viruses is approximately 1 week. This short incubation facilitates the virus to escape the immune response and cause the disease manifestations. Fever, head ache, rhinorrhoea and pneumonia are the common symptoms and create confusion when applying the treatment options. Emerging molecular-based detection methods have gradually replaced the conventional tests leading to the faster and reliable diagnosis. Drug development and vaccine design remain challenging due to the continuous evolution of these viruses. Hence, there are no specific drugs and vaccines (with complete reliable efficacy) to overcome these disease outbreaks; thus, preventive measures must be implemented to control infection.

For the past few years, World Health Organization, the Centre for Disease Control and Prevention, other Local Public Health organizations have worked using several strategies and guidelines to face challenges with these outbreaks. However, the underlying lacunae led to major epidemics and pandemics worldwide. In the earlier outbreaks of Influenza, SARS-CoV and MERS, public health authorities and clinical practitioners have learned lessons for managing these epidemics. The SARS-CoV in 2003 and MERS-CoV in 2012 subsequently affected worldwide and their air route transmission is leading to the development of control measures on air travel. After the emergence of SARS-CoV, WHO, International Health Regulation 2005 improvised with the recognition of Global Surveillance Systems and Epidemic Intelligence systems, such as the Global Public Intelligence Network and ProMed. Certain non-pharmaceutical interventions (NPIs) have been employed in the past and during the present COVID-19 pandemic. These include a mandatory face mask, quarantine, distancing, frequent hand wash, etc. Moreover, the International Severe Acute Respiratory and Emerging Consortium Infection (ISARIC) was launched in December 2011 as a step aiming to ensure that clinical practitioners and staff members get updated protocols as a rapid response to emerging outbreaks [169]. Concerning earlier experienced outbreaks of SARS-CoV, MERS-CoV and newly emerged coronavirus SARS-CoV2 (nCoV-19), general outbreak guidelines have been implemented to avoid contact transmissions. Rapid diagnostic availability would bring a high number of tests there by controlling the infection rate [170]. Following basic health hygiene such as washing hands, sanitization, gloves, gowns, and surgical masks avoid the risk of spreading the infection to health care attenders and other medical staff [171]. Air borne precautions are being implemented to prevent infection through the air or suspended particles. Keeping a distance of at least 1 m for avoiding contact transmission to healthy individuals [172]. Apart from these, social distancing prevents community transmission, which keeps healthy individuals away from symptomatic and asymptomatic patients. NPIs were also being practiced in few countries such as Japan during the non-covid time. Hence, these practices restricted the spread of COVID-19 and other respiratory viruses. Considering the current information regarding the respiratory viruses, we conclude that the characteristics of all the viruses be considered while carrying out the epidemiological surveillance and treatment execution.

## Supplementary Information

**Additional file 1: Table S1.** List of Respiratory Viruses and their major characteristics.

## Data Availability

All the data mentioned in this review article are available with the corresponding author.
