# Supplementary material for: Respiratory illness virus infections with special emphasis on COVID-19
Source: Eur J Med Res. 2022 Nov 8;27:236. doi: 10.1186/s40001-022-00874-x (PMC9641310; doi:10.1186/s40001-022-00874-x)
Supplement: Supplementary file 1 — Additional file 1: Table S1. List of Respiratory Viruses and their major characteristics. [file 40001_2022_874_MOESM1_ESM.docx]

| S.  **Suppl Table. 1 List of Respiratory Viruses and their major characteristics.**  NO. | VIRUS  NAME | TAXONOMIC  family | GENOME | PROTIEN  (Structural/Non structural) | ACCESSION  NO. | OTHER  DETAILS | Vaccine Availability |
| --- | --- | --- | --- | --- | --- | --- | --- |
| 1. | Adenovirus  A-F | *Adenoviridae* | dsDNA  Non-enveloped  34-36 Kb | E1A,  E1B,  E2,  E3,  E4,  IX,  IVa2,  UXP &MLTU | HAdv-C1  AC000017  HAdV-D  P33H27F67 | Mode of Transmission­: Inhalation of droplets containing viral particles.  Target Cells:  Upper or lower respiratory tract infection,  cornea,  lymphoid tissue | Yes ( live virus oral vaccine) |
| 2. | Human Bocavirus | *Parvoviridae* | -ssDNA  (~5kb) | 3 ORFs  Non-Structural:  NS1,  NP-1,  Structural:  Polymerase-VP1 &VP2 | DQ988934  GQ455988 | Mode of Transmission:  Inhalation of large aerosolized droplets of viral particles  Target cells:  respiratory epithelium,  lymphoid organs, CAR | No vaccine available |
| 3. | Influenza Virus (Swine flu, Avian influenza) | *Orthomyxoviridae* | -ss segmented RNA | Hemagglutinin (HA) Neuraminidase (NA) | AF333238; A/Brevig_Mission/1/18(H1N1 | Mode of Transmission:  Directly or indirectly touching infected person or objects touched by infected person  Target cells:  Upper or lower respiratory tract infection,  cornea,  lymphoid tissue | 1.Flu shot vaccine  2.Quadrivalent flu vaccine  3. Nasal spray  4.Adjuvanted  vaccine |
| 4 | Human Respiratory syncytial virus (HRSV) | *Paramyxoviridae* | -ssRNA  ~15kb | Structural: Nucleoprotein(N)  Glycoprotein(G)  Fusion(F)  SH (unknown)  RdRp(L)  Phosphoprotein(P)  M2-1  Non structural:  NS1, NS2, M2-2 | M11486  M17213 | Mode of Transmission:  Inhalation of aerosolized droplets of viral particles, or direct contact of droplets with ocular mucosa  Target cells:  alveolar epithelium, mononuclear cells | No vaccine available |
| 5 | Human parainfluenza virus (HPIV) type 1-4 | *Paramyxoviridae* | -ssRNA  Enveloped  15kb | Structural:  Nucleocapsid(L)  Glycoprotein(HN)  Fusion(F)  Membrane(M)  Non-Structural:  Zinc binding protein(V)  NS-C | 1)HPIV-1  KM190940  2)HPIV 2-  KY674949  MF077312  3)HPIV3-  KY973558  4)HPIV-4-  KY460518 | Mode of Transmission:  Inhalation of large aerosolized droplets of viral particles & Fomites  Target cells:  Cells of upper (URT)  and lower respiratory tracts (LRT) | No vaccine available |
| 6. | Human metapneumovirus (HMV) | *Paramyxoviridae* | -ssRNA Enveloped  ~13kb | Nucleoprotein(N)  Phosphoprotein(P)  Matrix protein(M)  Fusion Protein(F)  M2-1  M2-2 | AF371330  AF371367 | Mode of Transmission:­  Community Acquired, Inhalation of droplets containing viral particles  Target cells:  Lung epithelial cells | No vaccine available |
| 7. | Enteroviruses  A, B & C | *Picornaviridae* | +ssRNA  Non-Enveloped  7.4kb | 5` & 3` UTR  Structural –P1  Non-Structrual-P2 & P3 | KY425527 | Mode of Transmission­:  Fecal -Oral route  Target cells:  Coxsackievirus and adenovirus receptor (CAR) | 1.inactivated whole virus, 2. Recombinant VP1 protein, 3.Synthetic peptides, 4.Viral-like particles, 5.Live attenuated vaccines |
| 8. | Rhinovirus  A, B, C | *Picornaviridae* | +ssRNA  Env  7.4kb | UTRs (IRES)  Structural:  Capsid(P1)  Non-Structural:  P2  P3(RdRp) | Rhinovirus A  KX348029  Rhinovirus C-  JF17013  MG148341 | Mode of Transmission­:  Airborne, Hand to hand contact, and self-inoculation into eyes & nose  Target cells:  Respiratory epithelium,  ciliated &non ciliated cells of nasopharynx | No vaccine available |
| 9. | Human Corona Virus OC43,  229E,  HKCU1 | *Coronaviridae* | +ssRNA  Envelope  27-32kb  5`Capped  Poly A tail | Structural:  Spike(S),  Membrane(M),  Envelope(E),  Nucleocapsid(N)  Hemagglutinin(HE)  Non-structural:  NS2,NS4  RNP(ribonucleoprotein) | HCoV OC43  AY391777 | Mode of Transmission­:  Zoonotic origin, Hand to hand contact or by infected objects and self-inoculation into eyes & nose  Target Cells-airway epithelial cells, | No vaccine available |
| 10 | Severe Acute Respiratory Syndrome-Corona virus-1(SARS CoV-1) | *Coronaviridae*  *genus Betacoronavirus,* lineage B | +ssRNA  ~29.727kb  Enveloped | 14 ORFs,28proteins  5`UTR  M(Membrane protein)  N(Nucleocapsid protein)  S(Spike Glycoprotein)  HE (Hemagglutinin)  accessory proteins, known as ORFs 3a, 3b, 6, 7a, 7b, 8a, 8b, and 9b. | MN908947  AY274119 | Mode of Transmission:  Zoonotic origin(horseshoe bats)  Human to Human-Deposition of infected droplets or aerosols on the respiratory epithelium.  Target cells:  LRT, T-cell lymphoid organs. | No vaccine available |
| 11. | [Middle East respiratory syndrome coronavirus (MERS-CoV)](https://www.who.int/news-room/fact-sheets/detail/middle-east-respiratory-syndrome-coronavirus-(mers-cov)) | *Coronaviridae*  *genus Betacoronavirus,* lineage C | +ssRNA  ~ 30.119kb  Enveloped | 14 ORFs,10proteins  comprise two replicase polyproteins (ORF1ab and ORF1a),  four structural proteins (E, N, S, and M), and four non-structural proteins (ORFs 3, 4a, 4b, and 5) | NC_019843 | Mode of Transmission:  Zoonotic origin (horseshoe bats)  Human to Human-Deposition of infected droplets or aerosols on the respiratory epithelium.  Target cells:  LRT, T-cell lymphoid organs. | No vaccine available |
| 12. | SARS CoV-2 (Covid-19 disease) | *Coronaviridae* | +ssRNA  ~29.727kb  Enveloped | Structural:  Spike(S),  Membrane(M),  Envelope(E),  Nucleocapsid(N)  15 Non-structural proteins(NSP1-10 & NSP12-16) | [MN908947.3](https://www.ncbi.nlm.nih.gov/nuccore/MN908947.3) | Mode of Transmission­:  Zoonotic origin, Hand to hand contact or by infected objects and self-inoculation into eyes & nose  Target cells: airway epithelial cells, | Available:  Examples:  1.Covishield  2.Moderna&pfizer  3.Sputnik  4. Covaxin etc |
